# Traditional Chinese medicine for psoriasis vulgaris: A Protocol of a prospective, multicenter cohort study

**DOI:** 10.1097/MD.0000000000021913

**Published:** 2020-10-09

**Authors:** Xinwei Guo, Dongmei Zhou, Liyun Sun, Ping Wang, Jianhua Qu, Cang Zhang, Yan Wang, Zhaoxia Chen, Bo Li, Jing Hu, Zhimiao Lin, Fei Shi, Yanping Bai, Yuanwen Li, Xingwu Duan, Shentao Bao, Haibing Lan, Xiaoyan Sun, Xiong Wang, Xiang Liu, Linge Li, Litao Zhang, Fang Feng, Yujiao Meng, Qingwu Liu, Xiaoyao Guo, Jianning Guo, Yu Liu, Cong Qi, Jia Chen, Shuo Feng, Ping Li

**Affiliations:** a Beijing Hospital of Traditional Chinese Medicine, Capital Medical University, Beijing Institute of Traditional Chinese Medicine; b Beijing Hospital of Traditional Chinese Medicine, Capital Medical University; c Peking University First Hospital; d Air Force General Hospital, PLA; e China-Japan Friendship Hospital; f Dongfang Hospital Beijing University of Chinese Medicine; g Beijing University of Chinese Medicine Affiliated Dongzhimen Hospital; h Beijing University of Chinese Medicine Third Affiliated Hospital; i Beijing Hospital of Traditional Chinese Medicine Shunyi Branch; j Beijing Gulou Hospital of Traditional Chinese Medicine; k Beijing Daxing District People's Hospital; l Traditional Chinese Medicine hospital of Beijing Miyun; m Hebei Province Hospital of Traditional Chinese Medicine; n Traditional Chinese Medicine Hospital of Shijiazhuang City; o Tianjin Academy of Traditional Chinese Medicine Affiliated Hospital, dermatology department.

**Keywords:** cohort study design, long-term efficacy, psoriasis vulgaris, recurrence, traditional Chinese medicine

## Abstract

**Introduction:**

The incidence of psoriasis vulgaris is increasing worldwide. Chronic recurrence of the disease, as well as accompanying cardiovascular disease, metabolic syndrome, and depression has affected the physical and mental health of these patients. Psoriasis vulgaris is a difficult and major disease in the dermatology field. Short-term curative effects using conventional therapy for psoriasis vulgaris has made major strides. However, traditional Chinese medicine (TCM) treatment has long-term curative advantages for psoriasis vulgaris but lacks the scientific and clinical evidence for its use. This study intends to demonstrate and provide scientific and clinical evidence for the use of TCM to delay the recurrence of psoriasis vulgaris.

**Methods and analysis:**

This will be a prospective, multicenter cohort study. We intend to recruit 1521 psoriasis vulgaris patients from 14 hospitals in Beijing, Tianjin, and Hebei. Treatment will be based on the diagnosis specifications and clinical practice guidelines of TCM and conventional therapy. During inclusion and the subsequent follow-up period, doctors through electronic case reports will collect different therapeutic TCM regimens and conventional therapy that were administered. Information on life condition, skin lesions at each visit, World Health Organization Quality of Life Instruments, Zung Self-rating Anxiety Scale, Zung Self-assessment of Depression, laboratory examinations, incidence of new rash and recurrence during the remission and recurrence stages will be recorded.

**Ethics and dissemination:**

The clinical trial protocol for this study was approved by the ethics committee of the Beijing hospital of TCM affiliated to capital medical university (Ethics number: 2019BL02-010-02). We will publish and present our results at national and international conferences and in peer-reviewed journals specialized in dermatology.

**Trial registration:**

This protocol has been registered in clinicaltrials. gov (ChiCTR1900021629)

## 1 Introduction

Psoriasis is a common chronic, recurrent, immune-mediated dermatological disease. Environmental and genetic factors and their interactions are thought to contribute to the disease. Typical clinical manifestations are scaly erythema or plaques. They may be localized or present throughout the body. The disease is difficult to treat and often has lifetime risks. It affects about 3% of the worlds’ population, with a higher prevalence in Europe and the United States compared to China (0.123-0.47%). However, the incidence rates have been increasing over the years.^[1]^ Patients with psoriasis are usually accompanied with cardiovascular diseases, diabetes, metabolic syndromes, mental diseases and enteritis.^[2–4]^ It seriously affects the physical and mental health of patients and is a burden on medical resources. The 1-year recurrence rate of psoriasis is as high as 73.7%,^[5]^ and is a key concern for patients with psoriasis. High-risk factors for recurrence include tobacco use, alcohol, dampness, upper respiratory tract infections, certain medications, mental stress, being overweight and increased body fat ratios.

Conventional therapy that is used to treat psoriasis patients includes retinoic acid, phototherapy, immunosuppressive agents and targeted biological agents. However, these first-line therapies are expensive and have several adverse side effects.^[6,7]^ Previous studies have demonstrated that cyclosporin A, calcipotriol and methotrexate could moderately prolong psoriasis recurrence through sequential therapy, topical drug therapy, 308 excimer laser and biological agents .^[8]^ The majority of conventional therapies include a dosage reduction scheme, lifestyle changes and health education to delay recurrence. These include relaxation, behavioral changes, balanced diets, fish oils, and foods rich in n-3 fatty acid supplements, in addition to smoking and drinking cessation and the use of topical emollients.^[9–11]^ However, these measures do not prevent relapse. There is a critical need to find alternative therapies to prevent the recurrence of psoriasis. Latin American Clinical Practice Guidelines on the systemic treatment of psoriasis offers an updated therapeutic information and a reference for therapeutic decisions, with the main goal of improving patient care, controlling symptoms, and improving patients’ quality of life. However, it does not provide guidelines on how to prevent recurrence.^[12]^

Treatment of psoriasis using traditional Chinese medicine (TCM) has a long history. It has been shown to reduce recurrence and have long-term curative effects.^[13,14]^ TCM is considered as a supplement to achieve a healthy qi and improve immunity in the weak. It can alleviate clinical symptoms and improve cure rates. A qualitative study demonstrated that TCM treatment for psoriasis was safe and could prevent recurrence, however additional clinical studies were needed.^[15]^ Hence understanding TCM for the treatment of psoriasis vulgaris is the key to improving the short-term and long-term efficacy of psoriasis vulgaris. This study may provide evidence for determining the long-term curative effects of TCM for psoriasis vulgaris.

## 2 Methods and analysis

### 2.1 Study design

**Figure 1:**
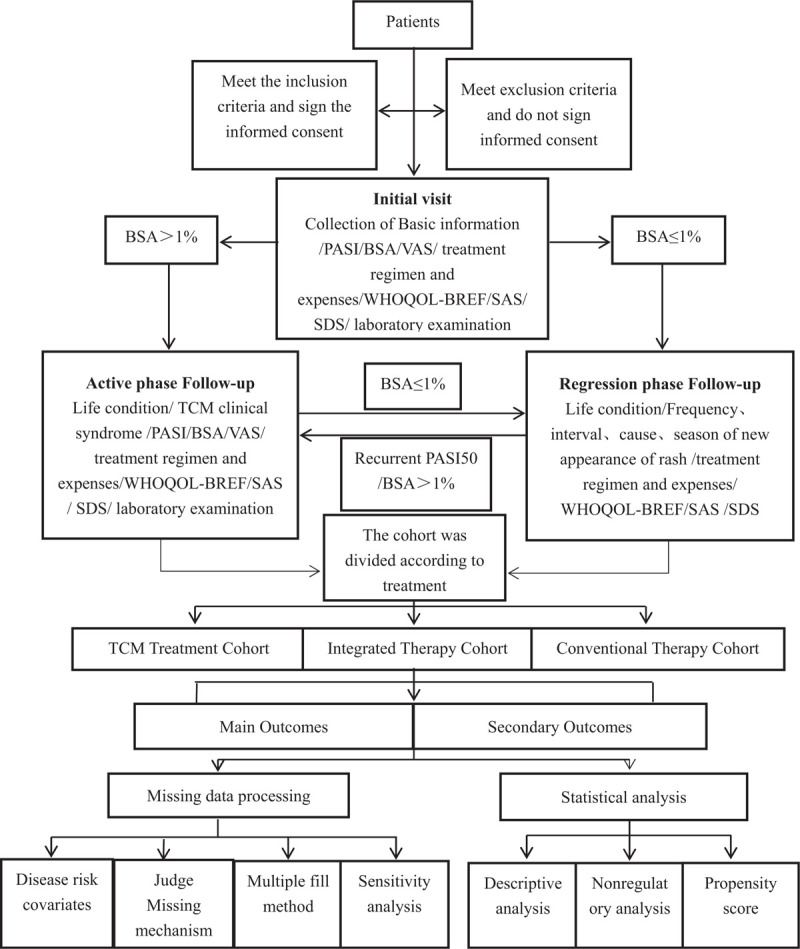
Flow Chart of the Study. BSA = Psoriasis surface area score, PASI = Psoriasis area and disease severity index score, SAS = Zung self-rating Anxiety Scale, SDS = Zung self-assessment of depression, TCM = traditional Chinese Medicine, VAS = visual analog scale of Psoriasis skin itch score, WHOQOL-BREF = World Health Organization Quality of Life Instruments. This figure described the flow chart of the Study.

This is a prospective, multicenter cohort study. The study was initiated in July 2019 and will end in December 2021. We plan to recruit 1521 psoriasis vulgaris patients from 14 hospitals in Beijing, Tianjin and Hebei province. All patients will be given therapies based on TCM and Conventional therapy guidelines and then followed-up for 1 year. Prior to recruitment, each participating hospital should be required to obtain ethics approval. Each participating hospital will follow a standard set of procedures. The flow chart of the study is depicted in Figure 1.

### 2.2 Study settings and participants

This study will be conducted in the following participating hospitals: Peking University First Hospital, Air Force General Hospital, PLA, China-Japan Friendship Hospital, Beijing University of Chinese Medicine Affiliated Dongzhimen Hospital, Dongfang Hospital Beijing University of Chinese Medicine, Beijing University of Chinese Medicine Third Affiliated Hospital, Beijing Hospital of TCM Shunyi Branch, Beijing Gulou Hospital of TCM, Beijing Daxing District People's Hospital, TCM hospital of Beijing Miyun, Hebei Province Hospital of TCM, TCM Hospital of Shijiazhuang City, and Tianjin Academy of TCM Affiliated Hospital.

### 2.3 Participants

#### 2.3.1 Eligibility criteria

The Inclusion criteria are as follows:

(1) Conform to the diagnostic criteria of Western and Chinese medicine for psoriasis vulgaris.^[16–18]^
(2) Study participants will not be selected or excluded until the required number of patients are met to ensure continuous enrollment.
(3) Volunteered to participate in the case registration study and signed an informed consent.

The Exclusion criteria are as follows:

(1) Patients with a combination of pre-existing serious systemic diseases, such as cardiovascular, liver, kidney and hematopoietic disease or other primary diseases.
(2) Severe mental illness.
(3) Patients with poor compliance, inability to communicate and unable to complete the pre-designed registration information.

#### 2.3.2 Patient cohort division

Patients received TCM will be classified as the TCM treatment cohort. The TCM treatment course should occupy more than half of the total course of treatment, with the following stratigies:

(1) oral herbal medicine once a day
(2) external use of Chinese medicine ointment with the area of body surface area (BSA) greater than or equal to the 10% of patients’ total BSA
(3) acupuncture treatment
(4) external use of TCM bath.

Patients received Conventional therapy following guidelines will be classified as the Conventional therapy cohort. Conventional therapies includ: external use of drugs (Cuticle promoter and Exfoliator pledge agent, glucocorticoid, vitamin D3, vitamin A acid derivatives, immunomodulatory drugs, and so on), Internal use of drugs (antibiotics, retinoic acid, glucocorticoid and immunosuppressants, biological agents, and so on). The treatment course should occupy more than half of the total course of treatment, with the following strategies:

(1) oral use of chemical drugs
(2) external use of chemical drugs ointment with the area of BSA greater than or equal to 10% of the total BSA
(3) biological agents injection.

Those who meet the requirements of TCM and Conventional therapy cohort meanwhile will be classified as the integrated therapy cohort.

Study assistants will be responsible for the initial visit and follow-up of each patient. The data collection schedule is shown in Table 1.

**Table 1:**
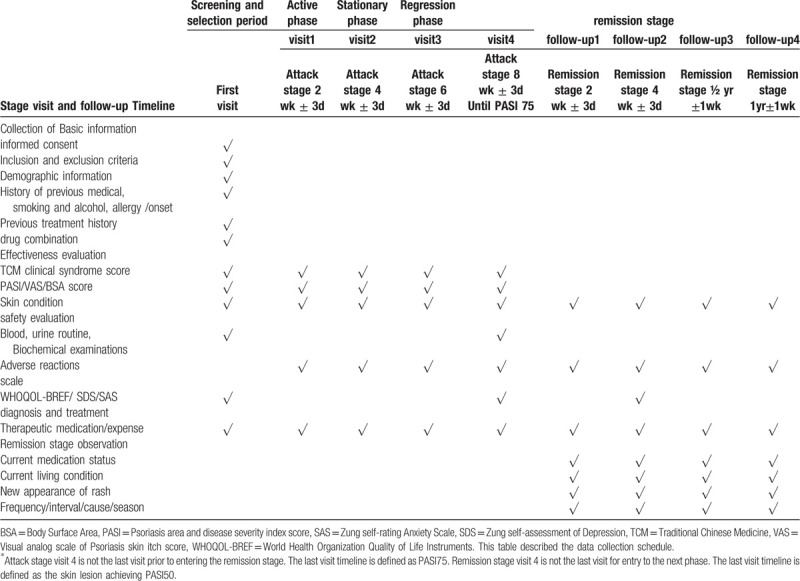
Data collection schedule.

### 2.4 Ethical approval

This study has been registered in clinicaltrials.gov (registration number ChiCTR1900021629) and was approved by the ethics committee of Beijing Hospital of TCM, Capital Medical University (Approval number 2019BL02-010-02). Patients who met the inclusion criteria will be enrolled after obtaining signed informed consent.

### 2.5 Outcomes

#### 2.5.1 Main outcomes

Recurrence rate in 6 months. Definition of recurrence: Patients have got relief psoriasis area and disease severity index score (PASI75), while the total PASI score increased to 50% of the original PASI.^[19]^

#### 2.5.2 Secondary outcomes

(1) Onset time: required time for 75% improvement of PASI scores
(2) Maintenance time: the time from improvement (PASI75) to recurrence (PASI50)
(3) BSA score: The patient's palm area equivalent to 1% of the BSA
(4) Visual analog scale of Psoriasis skin itch score (VAS) score: Urticant degree “0 minutes” represents “do not have urticant feeling”, increase in urticant gradually increased score values. 10 minutes represents “the most serious urticant score”.
(5) Skin relapse during treatment and follow-up period: The location of new skin lesions (head/trunk/arm/leg), number (1–2/3–5/6–10/>10) and new attack after 6-month interval (no attack/once, between 2-weeks to 1 month/once, every month to 2 months/once, every 2 to 3 months/once, every 3 to 4 months/once, after more than 5 months).
(6) World Health Organization Quality of Life Instruments, which included the 28 items, with full credit of 100
(7) Zung self-assessment of Depression, Depression was classified as either mild, moderate or severe.
(8) Zung self-rating Anxiety Scale (SAS), Anxiety level was classified as either mild, moderate or severe.
(9) The total score of the TCM clinical syndrome scale was calculated using blood heat syndrome, blood dryness syndrome and blood stasis syndrome.
(10) Treatment expenses (The cost of registration, TCM, Conventional therapy, examinations, physical therapy, acupuncture, transportation expenses, accommodation, and other expenses)

### 2.6 Safety evaluation

Safety evaluation was performed by observing adverse events (detailed records at any moment), in addition to evaluating symptoms, abnormal laboratory findings or other examinations (physical and laboratory examinations including blood routine, liver and kidney function, and biochemical examination). Adverse events will be identified by comparison, and causal analysis of adverse events will be performed according to “positively relevant,” “very likely relevant,” “probably relevant,” “probably irrelevant,” and “positively irrelevant.”

### 2.7 Sample size calculation

The sample size of this study was calculated based on the recurrence rates which is the primary outcome. According to the previous study, taking methotrexate as an example, the recurrence rate of conventional therapies is 73.7%,^[5]^ The recurrence rate of integrated Chinese Medicine is 28.6%,^[20]^ We suppose the recurrence rate of TCM group was 20%. Using non-equality test design by the PASS software, we set the α=0.05 and the power 1-β=0.8, the ratio of cases among each group is 1:1:1, the number of cases calculated is 390 in each group. We suppose a 30% of lost in the follow-up Of the study, the ultimately needed cases in each group is 507.

### 2.8 Data collection

We will use the “yiluyun clinical research information management system” as the data management platform. This database is designed by Beijing Institute of TCM, which data will be collected from PC, pad and phone. The hierarchy of users access can be ranked as entry clerk, supervisors and auditors.

The entered data can be returned for modification after submission, but the modification traces need to be kept. According to the Standard operating procedure for data entry, in the process of data entry, the person in charge of data entry shall guide in time and track the work progress and quality of data entry personnel. Once the data is submitted, the editing authority of this page will be cancelled.

### 2.9 Data validation

After data export, SAS9.4 software will be used to check the validation, which mainly refers to the following contents: conformation to the inclusion and exclusion criteria, existing of extreme values or logic errors for the measurement values of each visit, concomitant medication, and the occurrence of adverse events during the study.

### 2.10 Statistical analyses

#### 2.10.1 General principles

SAS 9.2 software will be used for statistical analyses. Qualitative indicators will be described using frequency tables, percentages or composition ratios and quantitative indicators will be described using mean, standard deviation, median, lower quartile (P25), upper quartile (P75), minimum value (min), and maximum value (max). The hypothesis test will be a 2-sided test and will include test statistics and their corresponding *P*-values. *P* <.05 will be considered statistically significant.

#### 2.10.2 Non regulatory analysis

For the comparative analysis of 2 groups, qualitative data will be analyzed using the chi-square test, Fisher exact probability, Wilcoxon rank-sum test and CMH chi-square test. Quantitative data in line with normal distribution will be analyzed using *t*-test (homogeneity test of variance between groups, test level at 0.05, and Satterthwaite method will be used for correction when the variance is uneven). Wilcoxon rank-sum test and Wilcoxon signed-rank sum test will be used when the data is abnormally distributed.

The survival analysis will be used to compare statistical significance between the groups (for the appearance of new skin lesions). In addition, the Cox regression model will be used to adjust the impact of baseline PASI values, course of the disease, and body mass index.

#### 2.10.3 Missing data analysis

Analysis of the impact of missing data will account for the loss of outcome variables. The data types gathered in this study will include longitudinal data that lists enrollment and completed cases for each center and the reasons for the missing data, as well as determining the distribution of each statistical set. We will then determine the mechanism of deletion, which may include the following 3 situations: complete random deletion, random deletion, and nonrandom deletion. For complete random deletion, the sample mean or general linear model will be used, for random deletion, the traditional regression model will be used, and for nonrandom deletion, the mixed-mode method will be used.

#### 2.10.4 Selection of covariates

Covariates will be selected from baseline data or other risk variables affected by treatment. Adjusted analysis will be performed for basic demographic characteristics, body mass index, course of the disease, medical history in the last 3 months, the co-occurrence of psoriatic arthritis, baseline PASI scores, SAS scores, Zung self-assessment of Depression scores, and so on. At the time of patient enrollment to the group. This is to determine if basic conditions between the groups are comparable. Other risk factors will include the use of western medicine (immunosuppressants, biological agents, hormones, antibiotics, and so on).

#### 2.10.5 Efficacy analysis (Adjustment analysis)

Efficacy analysis indicators will include changes in the main efficacy indicators from the initial visit (baseline) to each follow-up period.

Logistic regression analysis will be used to study the relationship between continuous dependent variables and independent variables. Influencing factors will be analyzed to determine which independent variable influences the probability of dependent variable occurrence. The decision analysis method will be used to estimate the probability of each category of the dependent variable (in the presence of a combination of various independent variables), to predict the outcome. The mixed effect of multiple covariates on treatment outcome will be adjusted using the propensity score matching method. First, we will determine if the distribution of patients with similar tendentiousness score is balanced among the groups. The distribution of patients will then be adjusted according to the tendentiousness score to calculate the effect estimation value. Stratified analysis will be conducted based on patients’ PASI score which will be divided into 4 groups based on the 4 levels of PASI score (less than 10,10–20,20–30 and more than 30).

#### 2.10.6 Safety index analysis

The safety index denotes the incidence of adverse events and abnormal changes in laboratory test results.

Adverse events will be listed based on the classification of adverse reactions and unrelated adverse events. Adverse reactions will include those that are “definitely related” and “possibly related” in this study.

Chi-square test or accurate probability method will be used to compare the incidence of adverse events between the 3 groups.

Changes in laboratory test results before and after treatment will be listed. Differences before and after standardization will be evaluated based on the normal value ranges for each central laboratory, in addition to the specific situation of the enrolled patient. Normality and abnormality will be determined using the normal value range table from each central laboratory and whether it has clinical significance.

## 3 Discussion

Psoriasis vulgaris has a high recurrence rate. The pattern of recurrence varies with each patient. Some patients will have early and frequent recurrence, while others may have long-term remission with few recurrences. Hence, patients may need maintenance treatment for an extended period of time. Psoriasis is characterized by long-term chronic course with recurrent attacks. RCTs that have been previously performed have not provided insights to preventing recurrence. However, real-world studies may have advantages to provide long-term insights. Previous publications have evaluated the clinical efficacy of biological agents for the treatment of psoriasis through retrospective real-world registration studies. However, no real-world or registration studies with regards to TCM for the treatment of psoriasis have been published in China.

This study did not include a placebo group, nor employed a blinded method. Patients in this study included elderly, children, pregnant and breastfeeding women and met the ethical requirements for enrollment in this study. With regard to the study design, we mainly focused on the Psoriasis Longitudinal Assessment and Registry^[21–23]^ (PSOLAR) and the National Central Cancer Registry of China^[24]^ (NCCR). The Psoriasis Registration Platform was designed to demonstrate, train researchers and to strictly adhere to standard operation process. In addition, it included guidelines for strict data collection and a management plan to improve data collection and transfer efficiency. The electronic case registration system is helpful to facilitate the interaction between doctors and patients. It actively monitors and collects information from diverse disease groups to improve data quality and authenticity. Data from the platform can also be easily shared. The platform meets the clinical needs and is reasonably standardize to facilitate the preservation of original medical records. These include access to BMI, PASI, and BSA indicators through an embedded formula. In addition, the platform contains drug library resources, diagnosis and treatment guidelines, to propose individualized prescriptions. Improvements in PASI, BSA and VAS scores for each patient's skin lesions can be visualized and monitored directly.

Based on an intelligent and information-based data collection and data management platform, this study observed and followed-up on thousands of patients within a short period. Data will be analyzed after centralized integration to compare conventional therapy with that of the combination of Chinese and Western medicine treatment. Patients will be evaluated based on recurrence rate 6 months after PASI75, onset time, maintenance time, the incidence of new rashes during treatment and follow-up, World Health Organization Quality of Life Instruments/BSA/VAS scores and treatment costs. The long-term efficacy of TCM in treating psoriasis vulgaris through intelligent and information-based data collection and management will then be established and evaluated. It determined the advantages of TCM and provided real-world evidence for the long-term efficacy of TCM for the treatment of psoriasis. In addition, the platform could evaluate the mode and cost-effectiveness of psoriasis vulgaris treatment in the real world. Improvement of skin lesions, patient quality of life, recurrence rates of skin lesions and the state of anxiety and depression will also be able to be evaluated in the future. Long-term follow-ups of patients during remission and recurrence will provide clues to inducing factors and effective interventions for patient treatment. Using the multicenter registration platform to collect large amounts of standardized data within a short period will help mine and analyze long-term efficacy data of TCM for the treatment of psoriasis from the perspective of recurrence.

Patient management, collection, and analysis of medical data is the aim of the present clinical study. Management of chronic diseases will be investigated using this methodology. Patients will be initially registered using unique codes and then will undergo the relevant examinations based on an standard operation process to ensure consistency. Second, patients will be regularly evaluated for their disease condition by regular follow-up visits. If patients are unable to be physically present, they will be evaluated via online or telephone follow-ups. Third, doctors and patients will have direct access via Wechat IDs managed by research assistants. For common problems, the research assistants will communicate with patients via Wechat to improve patient compliance. Fourth, the database will be regularly checked. Patient treatment plans often change, so it is necessary to review and modify information regularly. Treatments will be evaluated for efficacy and actual benefits of TCM for chronic diseases. After platform establishment, enrollment of patients based on inclusion and exclusion criteria should be strictly followed. Based on preset objectives, there will be different research plans and corresponding statistical analysis required. It will be necessary to hold regular meetings to further develop the platform, share knowledge and data so that investigators can benefit from this platform.

## Acknowledgments

We thank all the team members who participated in this study. The authors also thank all the patients.

## Author contributions

XWG wrote the first draft of the article of the study protocol, participated in the Platform construction, research design, performed project, quality control, enrolled patients and datas collection. PL was responsible for writing-review and editing, developing the study protocol, conducting and supervising the clinical study as a primary investigator. SF contributed to the design of the study, supervised the study protocol and revised the manuscript. DMZ, LYS, PW, JHQ, CZ, ZML, FS, YPB, YWL, XWD, STB, HBL, XYS, XW, XL, LEL, LTZ, YW and ZXC conceived the study and enrolled patients. FF, YJM, QWL, XYG, JNG, YL, CQ and, JC are responsible for datas collection. BL and JH are responsible for datas analysis. All authors have contributed to the design and implementation of the study. All authors read and approved the final manuscript.

## Abbreviations

Abbreviations: BSA = body surface area, PASI = psoriasis area and disease severity index score, SAS = Zung self-rating Anxiety Scale, TCM = traditional Chinese medicine, VAS = visual analog scale of Psoriasis skin itch score.
